# Punctate Inner Choroidopathy

**DOI:** 10.1155/2015/371817

**Published:** 2015-04-28

**Authors:** Mariana Sá-Cardoso, Arnaldo Dias-Santos, Natália Nogueira, Heloísa Nascimento, Rubens Belfort-Mattos

**Affiliations:** ^1^Department of Ophthalmology, Centro Hospitalar do Baixo Vouga, Aveiro, Portugal; ^2^Department of Ophthalmology, Centro Hospitalar de Lisboa Central, Lisbon, Portugal; ^3^Department of Ophthalmology, Universidade Federal de São Paulo (UNIFESP), São Paulo, SP, Brazil

## Abstract

*Purpose*. To report a case of bilateral punctate inner choroidopathy (PIC). *Case Report*. A 26-year-old Caucasian woman presented with bilateral blurred vision with one year of evolution. There was no relevant systemic disease or family history. Best-corrected visual acuity in the right eye was 20/30 and in the left eye was 20/20; there was no clinically significant refractive error. Fundoscopy evidenced multiple, small, round, yellow-white lesions limited to the posterior pole of both eyes, with greater macular involvement in the RE. There were no signs of inflammation in the anterior chamber or vitreous cavity. Fluorescein angiography revealed the presence of multiple hyperfluorescent lesions more evident in the later stages of the angiogram in both eyes. On indocyanine green angiography, these lesions appeared hypofluorescent in both early and late phases. Optical coherence tomography showed the presence of focal elevations of the retinal pigment epithelium with underlying hyporeflective space, bilaterally. Laboratory and imaging evaluation for evidence of autoimmune and infectious diseases were negative. *Conclusion*. The PIC is a relatively uncommon condition. In this report, an attempt has been made to describe a classic clinic presentation of this disease in a young and female patient.

## 1. Introduction

Punctate inner choroidopathy (PIC) is a relatively uncommon inflammatory multifocal chorioretinopathy that affects predominantly young myopic women [1–4]. This disease is characterized by the development of multiple, small (100–300 *μ*m), yellow-white spots in the posterior pole of each eye, at the level of the inner choroid and retinal pigment epithelium (RPE), which occurred in the absence of clinically apparent intraocular inflammation. They are often associated with small serous neurosensory retinal detachments. After a few weeks, these acute PIC lesions resolve, leaving atrophic spots with variable pigmentation [3]. It was first described and named by Watzke et al. [4] in 1984, when they reported a series of 10 myopic women who presented with blurred central vision, flashes of light, and paracentral scotomas. Eight of the 10 presented with bilateral lesions and 6 developed choroidal neovascular membranes (CNV). No patient had flare or inflammatory cells in the anterior chamber or vitreous cavity. An accurate estimation of the incidence and prevalence of PIC is difficult due to the wide spectrum of ocular involvement and its visual sequelae. In a recent series [2], 90% of patients affected by PIC were women and 97% were Caucasian, with a median age at presentation of 30 years (range 15–55). Most (85%) were myopic, with median prescription strength of −7.00 D in each eye (range −1.25 to −12.75 D); only 1% were hyperopic. In many patients, PIC is a self-limited disease with good visual prognosis. However, in approximately 40% of patients, more severe visual loss can occur as a result of the development of CNV [1, 3]. No treatment is advised for the majority of patients with PIC where there is no evidence of CNV. Nevertheless, the therapeutic modalities for PIC-related CNV are not well established, but reported treatment regimens often include corticosteroids, PDT, and/or anti-VEGF molecules [1, 2].

## 2. Case Report

A 26-year-old Caucasian woman presented with bilateral blurred vision with one year of evolution. There was no relevant systemic disease or family history.

**Figure 1:**
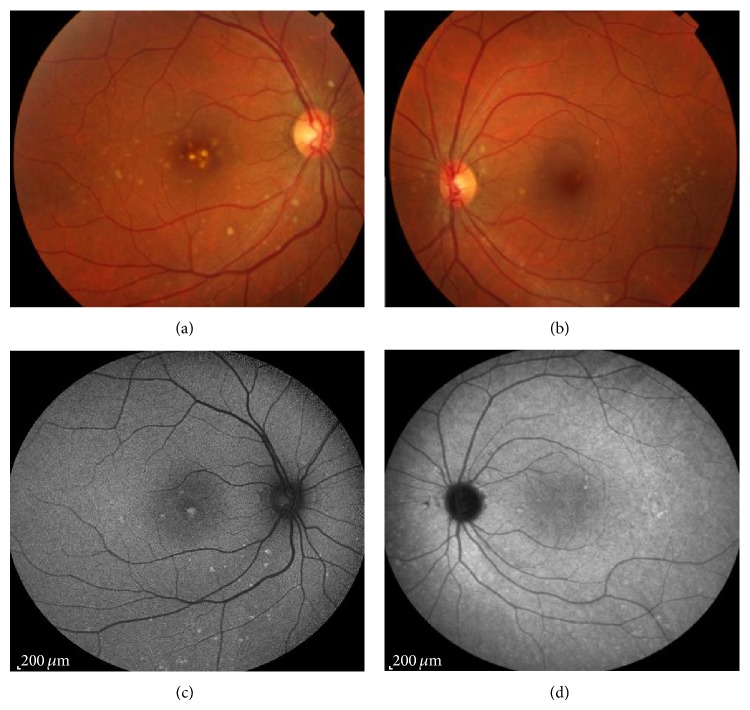
Retinography evidenced multiple, small, round, yellow-white lesions limited to the posterior pole of both eyes, with greater macular involvement in the RE.

**Figure 2:**
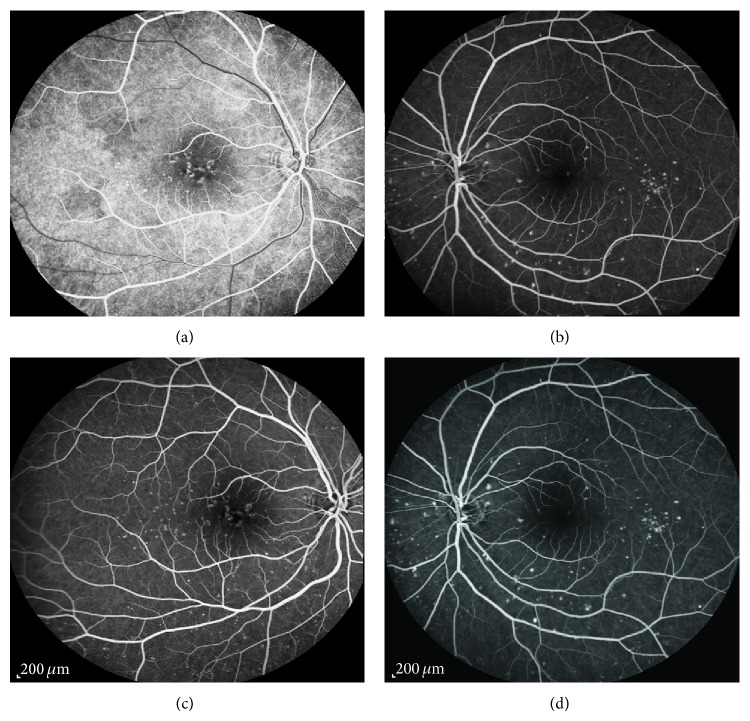
Fluorescein angiography revealed the presence of multiple hyperfluorescent lesions more evident in the later stages of the angiogram in both eyes.

**Figure 3:**
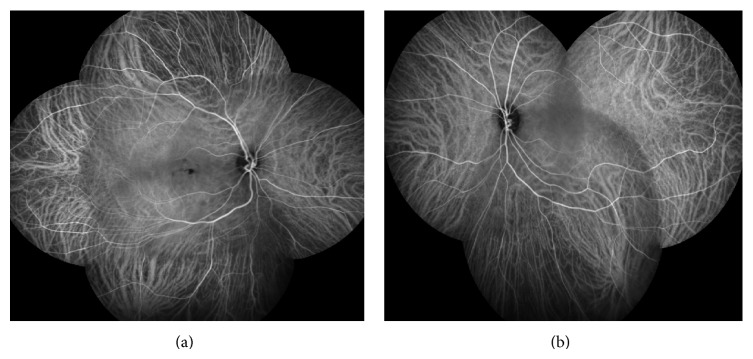
Indocyanine green angiography (ICG) revealed hypofluorescent lesions in both early and late phases, more evident in the RE.

**Figure 4:**
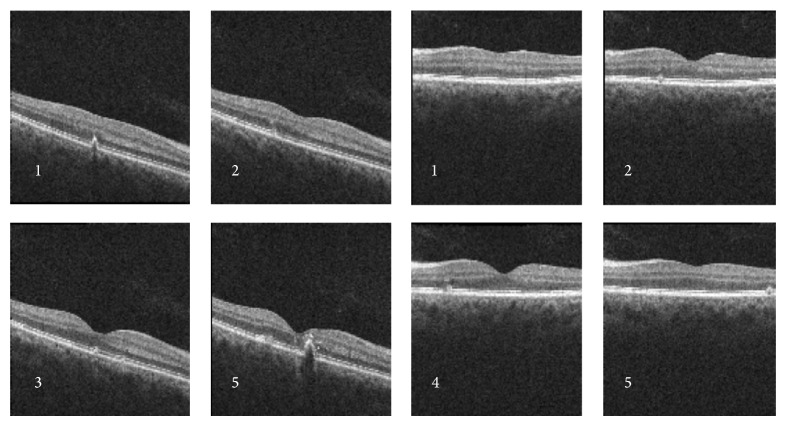
Optical coherence tomography showed the presence of focal elevations of the RPE with underlying hyporeflective space, bilaterally.

Best-corrected visual acuity (BCVA) in the right eye (RE) was 20/30 and in the left eye (LE) was 20/20; there was no clinically significant refractive error. Fundoscopy evidenced multiple, small, round, yellow-white lesions limited to the posterior pole of both eyes, with greater macular involvement in the RE (Figure 1). There were no signs of inflammation in the anterior chamber or vitreous cavity. Fluorescein angiography (FA) revealed the presence of multiple hyper fluorescent lesions more evident in the later stages of the angiogram in both eyes (Figure 2). On indocyanine green angiography (ICG), these lesions appeared hypofluorescent in both early and late phases (Figure 3). Spectral-domain optical coherence tomography (SD-OCT) showed the presence of focal elevations of the RPE with underlying hyporeflective space, bilaterally (Figure 4). Laboratory and imaging evaluation for evidence of autoimmune and infectious diseases were negative. In light of these findings, we diagnosed punctate inner choroidopathy and regular follow-up visits were scheduled. Six months after presentation, her visual acuity remained the same but the blurred vision complaints had resolved. Fundoscopic, FA, and OCT findings did not change significantly and there was no sign of CNV development.

## 3. Discussion

Punctate inner choroidopathy is a relatively uncommon ocular inflammatory disease that affects primarily young, myopic, and Caucasian women. Nevertheless, it can occur among men, non-Caucasians, and emmetropes or hyperopes [2]. We presented a case of a young and Caucasian female patient.

Visual acuity (VA) at presentation is often good in PIC [1, 4]. In the study realized by Watzke and colleagues [4], the majority of eyes (66.7%) had VA of 20/50 or better. Similar findings were reported by Reddy and colleagues [5] in their series of 16 patients, where over 75% had VA of 20/40 or better. Brown Jr. et al. [6] reported that 88% of patients with PIC had bilateral disease. Our patient manifested the disease bilaterally and her BCVA was ≥20/30, which is in agreement with the results obtained in the studies mentioned before.

Diagnosis of PIC can be difficult because the appearance may be similar to other conditions and types of posterior uveitis, especially other forms of the so-called white dot syndromes. It is very important to differentiate multifocal choroiditis and panuveitis (MCP) from PIC, because the management is significantly different. The difference is the presence of vitritis and/or iritis in patients with MCP. Acute posterior multifocal placoid pigment epitheliopathy (APMPEE) is differentiated by the level and size of the lesions, which are slightly more superficial and larger than those in PIC. On FA, APMPPE shows characteristic early hypofluorescence of the lesions in contrast to the hyperfluorescent lesions in PIC. Diffuse subretinal fibrosis (DSF) syndrome can mimic advanced stages of PIC; the difference is that, in DSF, the course is much more rapidly progressive and carries a worse prognosis. Birdshot retinochoroidopathy presents with midperipheral and peripheral retinal lesions. Multiple evanescent white dot syndrome presents with lesions at the level of RPE, which usually resolve without leaving scars or CNV, unlike PIC [1].

The lesions of PIC appeared to be located at the level of the choroid and RPE. Imaging studies have revealed leakage of active lesions on FA and evidence of involvement of choriocapillaris on ICG [1, 7, 8]. The most commonly observed morphology of lesion on SD-OCT involved RPE elevation with sub-RPE signals and the photoreceptors appear to be compressed during RPE elevation, which can explain the clear visibility upon resolution of the RPE changes [3, 7, 8]. The results of the ancillary tests realized in our patient enabled us to make the diagnosis of this pathology.

The most severe vision-threatening complications of PIC are choroidal neovascularization and subretinal fibrosis. Therefore, early diagnosis and close monitoring for development of choroidal neovascularization may benefit patients with PIC so that management of this complication can be initiated immediately [2].
